# Superfast Synthesis of Stabilized Silver Nanoparticles Using Aqueous *Allium sativum* (Garlic) Extract and Isoniazid Hydrazide Conjugates: Molecular Docking and In-Vitro Characterizations

**DOI:** 10.3390/molecules27010110

**Published:** 2021-12-24

**Authors:** Jamal Moideen Muthu Mohamed, Ali Alqahtani, Thankakan Vimala Ajay Kumar, Adel Al Fatease, Taha Alqahtani, Venkatesan Krishnaraju, Fazil Ahmad, Farid Menaa, Ali Alamri, Ranjini Muthumani, Rajendran Vijaya

**Affiliations:** 1 College of Pharmacy, Shri Indra Ganesan Institute of Medical Science, Manikandam, Tiruchirapalli 620012, Tamil Nadu, India; jmuthumohamed@gmail.com; 2 Department of Pharmacology, College of Pharmacy, King Khalid University, Guraiger, Abha 62529, Saudi Arabia; amsfr@kku.edu.sa (A.A.); ttaha@kku.edu.sa (T.A.); krishcology@gmail.com (V.K.); 3 Azidus Laboratories Ltd., Rathinamangalam, Chennai 600048, Tamil Nadu, India; ajayngl2000@gmail.com; 4 Department of Pharmaceutics, College of Pharmacy, King Khalid University, Guraiger, Abha 62529, Saudi Arabia; afatease@kku.edu.sa (A.A.F.); aamri@kku.edu.sa (A.A.); 5 Department of Anesthesia Technology, College of Applied Medical Sciences in Jubail, Imam Abdulrahman Bin Faisal University, Jubail P.O. Box 4030, Saudi Arabia; fmahmad@iau.edu.sa; 6 Department of Nanomedicine, California Innovations Corporation, San Diego, CA 92037, USA; dr.fmenaa@gmail.com; 7 Department of Pharmaceutical Technology, BIT Campus, Anna University, Tiruchirappalli 620024, Tamil Nadu, India; rajinimuthumani@gmail.com

**Keywords:** green synthesis, nanoconjugates, silver nanoparticles, isoniazid hydrazide, garlic extract, molecular docking

## Abstract

Green synthesis of silver nanoparticles (AgNPs) was synthesized from fresh garlic extract coupled with isoniazid hydrazide (INH), a commonly used antibiotic to treat tuberculosis. A molecular docking study conducted with the selected compounds compared with anthranilate phosphoribosyltransferase (trpD) from *Mycobacterium tuberculosis*. The aqueous extract of garlic was prepared and mixed with silver nitrate (AgNO3) solution for the superfast synthesis of stable AgNPs. INH was then conjugated with AgNPs at different ratios (*v/v*) to obtain stable INH-AgNPs conjugates (AgNCs). The resulting AgNCs characterized by FTIR spectra revealed the ultrafast formation of AgNPs (<5 s) and perfectly conjugated with INH. The shifting of λmax to longer wavelength, as found from UV spectral analysis, confirmed the formation of AgNCs, among which ideal formulations (F7, F10, and F13) have been pre-selected. The zeta particle size (PS) and the zeta potential (ZP) of AgNPs were found to be 145.3 ± 2.1 nm and −33.1 mV, respectively. These data were significantly different compared to that of AgNCs (160 ± 2.7 nm and −14.4 mV for F7; 208.9 ± 2.9 nm and −19.8 mV for F10; and 281.3 ± 3.6 nm and −19.5 mV for F13), most probably due to INH conjugation. The results of XRD, SEM and EDX confirmed the formation of AgNCs. From UV spectral analysis, EE of INH as 51.6 ± 5.21, 53.6 ± 6.88, and 70.01 ± 7.11 %, for F7, F10, and F13, respectively. The stability of the three formulations was confirmed in various physiological conditions. Drug was released in a sustainable fashion. Besides, from the preferred 23 compounds, five compounds namely Sativoside R2, Degalactotigonin, Proto-desgalactotigonin, Eruboside B and Sativoside R1 showed a better docking score than trpD, and therefore may help in promoting anti-tubercular activity.

## 1. Introduction

Multiple drug resistance (MDR) is one of the major concerns worldwide in the treatment of microbial infections. For instance, tuberculosis (TB), caused by the emergence of resistant *Mycobacterium tuberculosis* (MTB) strains, represents a global challenge [1].

Garlic can be used as a natural adjuvant to conventional treatments against the burden of MDR-TB pandemic [2,3]. Direct conjugation of isoniazid hydrazide (INH) with a weed extract of garlic by simple incubation, was effective against MDR-TB strains in vitro luciferase reported phage (LRP) assay [4]. In other in vitro studies (e.g., broth microdilution assays), aqueous extract of garlic and its combination with INH effectively inhibited MDR strains of TB [5]. Furthermore, green synthesized Silver nanoparticles (AgNPs) from garlic extracts were found stable for a year enhanced antibacterial activity and were thereby suggested in the treatment of TB [6]. Generally, the therapeutic value of garlic remains in its geographical source and the stability of the active component, namely allicin, which is rapidly converted to other compounds [7].

Considering the anti-TB potential of garlic extract, the high stability of AgNPs synthesized from garlic extract, as well as the effectiveness of INH combined to aqueous garlic extract against MDR-TB, an attempt has been made to formulate INH nanoconjugates using AgNPs from aqueous garlic extract. The established nanoconjugate (NC) drug delivery has been approved by FDA and come into the phase of clinical trials, offering a scope for multidrug treatment for tuberculosis.

Metal nanoparticles are a new category of materials that are now being studied for biological and therapeutic applications. Gold, silver, titanium oxide, iron nanoparticles, and other metal nanoparticles are the most commonly studied metal nanoparticles, and they have numerous applications in biomedicine. Metallic nanoparticles, in general, have a lot of potential in diagnostic imaging and targeted drug administration [8]. They are employed in medication therapy to improve the therapeutic activity of drug through active and passive targeting mechanisms. They eventually lessen the medications’ harmful adverse effects on normal tissues. They have the ability to transport large amounts of drugs and deliver them to the targeted location. [9]

Environmentally friendly approaches in chemistry and chemical technology are becoming increasingly popular, and they are critically needed in light of the widespread difficulties associated with pollution. As a result, due to its simplicity, non-toxic nature, cost effectiveness and eco-friendliness, the employment of green technology in nanoscience research has been a popular choice in recent years [10].

The green synthesis of nanoparticles from metal salts can be accomplished in a variety of techiniques. Microorganisms, plant extracts, whole plants, phytoconstituents, vitamins, and biodegradable polymers can all be used to synthesis nanoparticles. Plants produce a variety of metallic nanoparticles using hytochemicals, which are long-lasting and environmentally friendly reservoirs. In the synthesis of nanoparticles, plant extracts and phytochemicals may act as both reducing and stabilising agents [11,12].

Molecular docking is a well-organized technique and consists of calculating the leading binding modes of the ligand and a three-dimensional structure of a protein [13]. The prediction of binding modes (way by which the binding occurs) is crucial to reveal key structural characteristics, interactions, and permits supportive statistics in designing successful inhibitors [14]. The most attractive interaction applied in medicine is the protein and ligand interaction [15]. A ligand is known as a small molecule, and with its various possible conformations, it can interact with the binding sites of a protein drug design, the molecular docking is habitually utilized to recognize the drug information and its receptor interactions. It is recurrently used to find the binding orientation, affinity, and the activity of drug candidates towards a protein of target [16]. The docking study was used here to evaluate the preliminary assessment of the binding affinity of garlic derivatives towards the protein anthranilate phosphoribosyltransferase (trpD).

In this study, several prepared AgNPs and INH nanoconjugates were physically characterized in vitro and assessed for their toxicity ex-vivo. Furthermore, earlier reported compounds isolated from the garlic extract were evaluated by docking studies for their anti-tubercular potency.

## 2. Results and Discussion

### 2.1. Phytogenic Preparation of INH-Loaded AgNPs (AgNCs)

The formation of AgNCs were confirmed using λmax obtained by UV-Vis spectrophotometry for the various compositions (*n* = 13). The inter-analysis of INH/AgNPs microvolumetric ratio (*v/v*) corresponding to 400/600 (F7), 700/300 (F10), and 1000/1000 (F13) showed consistent λmax values (*p* < 0.05), so these three formulations were chosen for further characterizations. Indeed, such formulations have been considered as the most stable in previous studies [17].

**Figure 1:**
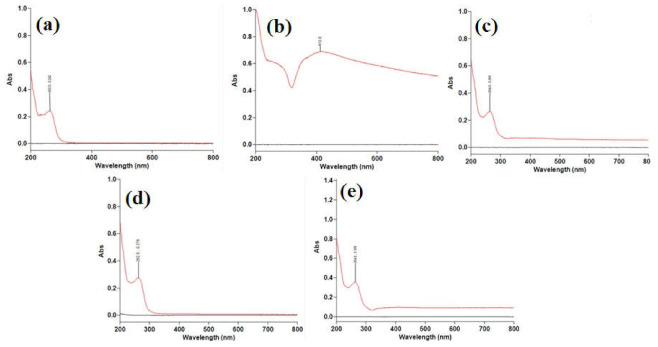
UV-Visible spectrum in the range 200–800 nm of (**a**) INH, (**b**) AgNPs, (**c**) F7, (**d**) F10, and (**e**) F13 λmax is indicated in each spectrum.

The UV-Vis spectra of INH, AgNPs, and the nanoconjugates F7, F10, and F13, are provided in Figure 1. The INH and AgNPs displayed λmax at 263 nm (Figure 1a) and 425 nm (Figure 1b), respectively. The AgNPs were yellowish-brown, which agree with other studies [18]. The UV absorption by colloidal AgNPs lies in the region of 400–450 nm is more likely due to the excitation of surface plasmon resonance/vibration (SPR) effect. AgNCs displayed λmax of 264 nm (Figure 1c–e). There were no other peaks found in the UV spectrum of AgNCs, indicating that the conjugation of INH with AgNPs occurred adequately. Hence, F7, F10 and F13 were definitively chosen for further analyses.

### 2.2. Drug Entrapment Efficiency (EE) and Loading Capacity (LC) of AgNCs

**Figure 2:**
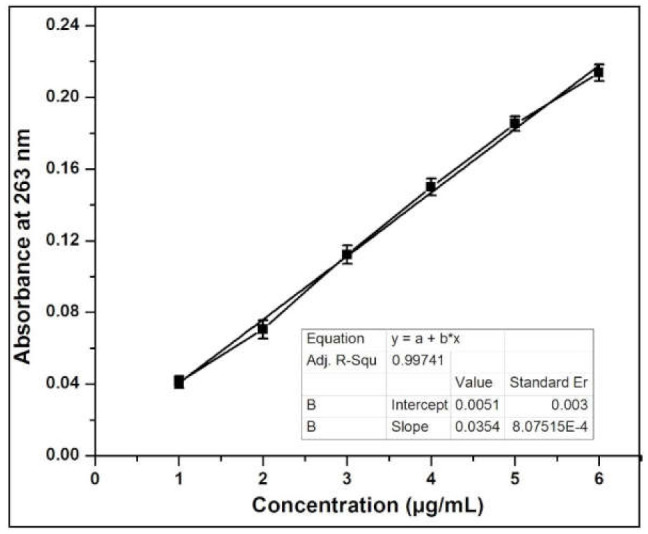
Calibration graph of INH at λmax 263 nm.

A calibration plot of INH was carried out at λmax of 263 nm with a R^2^ value of 0.9974 (Figure 2), and the INH content in AgNCs was measured using UV-Vis spectrophotometry. The results showed that the drug loading gets increased with increasing concentration of INH and AgNPs. The concentration-dependent loading of INC into AgNPs causes an increase in the size of AgNCs. The percentage of EE of INH into AgNPs was 51.6 ± 5.21%, 53.6 ± 6.88%, and 70.01 ± 7.11% for F7, F10 and F13, respectively. The percentage of LC of INH into AgNPs was 22.7 ± 2.54%, 28.18 ± 2.78% and 34.65 ± 3.33% for F7, F10 and F13, respectively. The dispersed phase capacity plays the major role on AgNPs, which progressively improved EE and LC [19]. The results show that by reducing the volume of the dispersed phase, the medium increased the viscosity of the dispersed phase, allowing for faster solidification and less INH leakage into the continuous phase. The percentage of EE and LC are in good agreement with the previous work reported by El-Say [20].

### 2.3. Physical Characterizations

#### 2.3.1. Interactive Functional Chemical Groups by FTIR Spectroscopy

**Figure 3:**
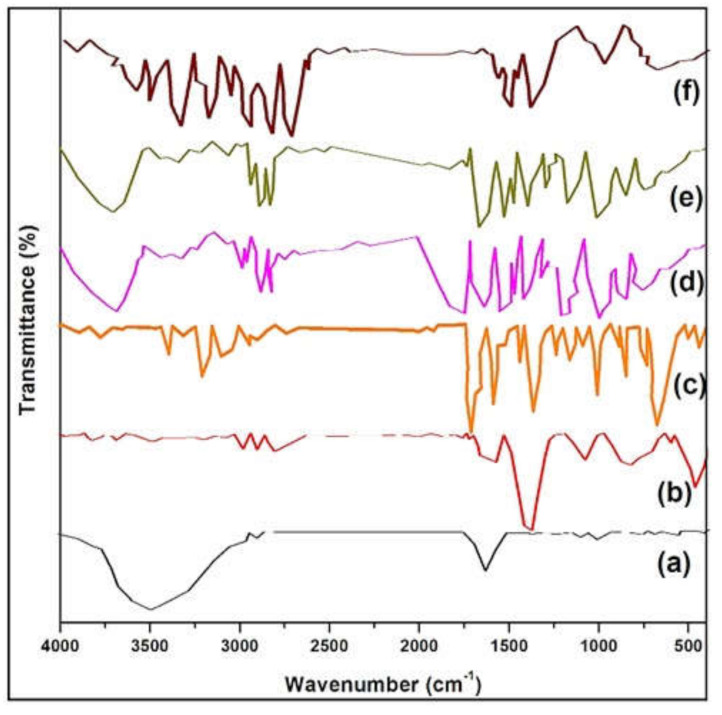
The FTIR spectrum of (**a**) Garlic extract, (**b**) AgNPs, (**c**) INH, (**d**) F7, (**e**) F10, and (**f**) F13.

The FTIR spectra of garlic extract, AgNPs, INH, and AgNCs (i.e., F7, F10 and F13) revealed no incompatibility among the formulation ingredients, as observed from the functional group peaks of these spectra (Figure 3).

The FTIR of aqueous garlic extract (Figure 3a) exhibited a peak at 1644.02 cm^−1^, which is assigned to carbonyl or carboxylic acid (C=O) stretching of peptide (amide) linkages, while the peaks at 1132.97 cm^−1^ and 1032.69 cm^−1^ were due to SO_2_ absorption of sulphones and primary amines, respectively. The peak at 1407.78 cm^−1^ reflects the amide II band. These data are in accordance with earlier reports [21].

The FTIR spectrum of AgNPs (Figure 3b) showed hydroxyl group (O-H) vibrations at 2962.13 and 2885.95 cm^−1^, suggesting the reduction and capping of AgNPs due to flavonoids and proteins in the garlic extract.

The FTIR spectrum of INH (Figure 3c) showed a strong amide band of C=O stretch vibration at 1669 cm^−1^, N-H bend at 1554 cm^−1^, and pyridine band at 1409.71 cm^−1^ with free NH_2_ at 1217 cm^−1^. These data are in accordance with previously reported data [22].

The phytogenic prepared AgNCs exhibited the asymmetric stretching vibration of INH carboxylic group, which is observable at 1557.24 cm^−1^ for F7, at 1555.31 cm^−1^ for F10, and at 1550.49 cm^−1^ for F13 (Figure 3d,f). The protonated amino group of INH was observed between 3235 cm-1 and 3113.51 cm^-1^. It confirmed the loading of INH into AgNPs [23].

The wavenumbers of each spectra and the characteristic of functional bands obtained are given in Table 1.

#### 2.3.2. Elemental Analysis by EDX

The formation of AgNPs was further confirmed by EDX (Figure S1 in the Supplementary Materials). EDX data revealed the chemical purity of the prepared AgNPs, which exhibited strong signal for the silver atoms. Additional peaks of C, O, and Cl biomolecules were present on the surface of AgNPs. The phytogenic AgNPs showed strong absorption in the range of 2.5–4 Kev. Similar results, obtained in the range of 2–4 Kev, have been reported earlier when AgNPs where synthesized using *Artemisia nilagirica* leaf or *Artocarpus heterophyllus* seed extracts [24].

#### 2.3.3. Crystallinity Nature by XRD

**Figure 4:**
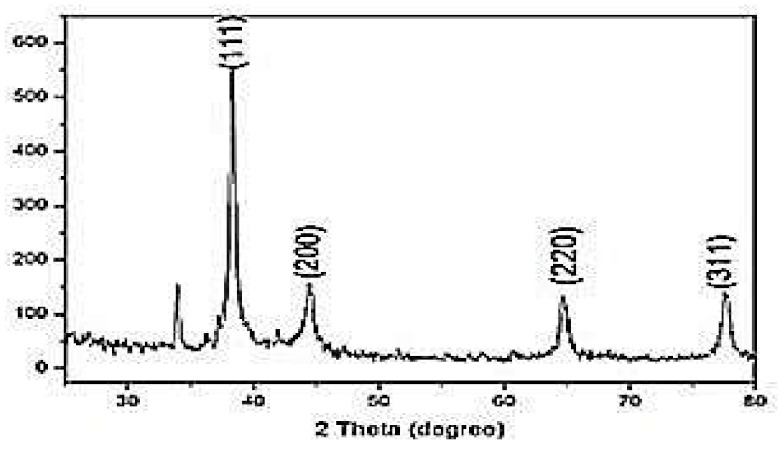
XRD spectrum of AgNPs synthesized from Allium sativum (garlic).

XRD diffraction peaks obtained at 38.35 o, 44.52 o, 64.17 o and 77.65o (2θ) were assigned to the (111), (200), (220) and (311) planes of a faced center cubic lattice of silver (Figure 4). According to the Muniz et al., the PS increases only the peaks at greater 2θ angles provide good results, and if one uses peaks with 2θ > 60 the limit for use of the Scherrer equation would go up to 1 mm [25]. The peak with high intensity reflects the degree of crystallinity of the AgNPs. The broader diffraction peaks further indicate the smaller crystallite size. It can be notice that there is one unassigned weaker peak of Silver that appeared at 33.89 o. These results are in accordance with previous studies [26], confirming the crystallinity nature of the prepared phytogenic AgNPs.

#### 2.3.4. Surface Morphology by TEM and SEM

**Figure 5:**
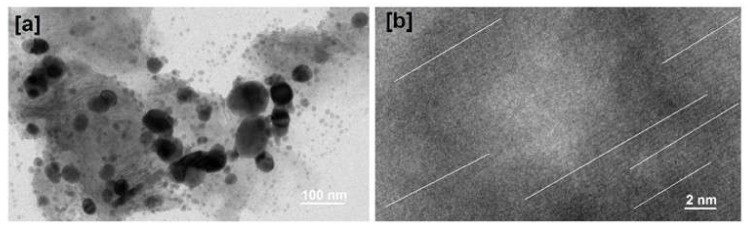
HR-TEM images of AgNCs at (**a**) bar scale of 100 nm, (**b**) bar scale of 2 nm.

TEM micrographs of AgNCs (Figure 5a,b) depicted a quasi-spherical structure with an average PS of 170 nm (Figure 5a). The structure of AgNCs was connected in a longitudinal and close pattern, and the attached or unentrapped mass was not seen on the surface of the NCs (Figure 5b). These observations clearly indicate that the origination of INH might be closely complexed with surface of the AgNPs similarly to what was observed by Wang and their team [27].

SEM micrographs of AgNCs, obtained at different magnifications (Figure S2a,b), depicted agglomerated spherical AgNCs (Figure S2a) with an average size ranging from 162 to 281 nm (according to the formulation), which were expectedly thicker than the average size of AgNPs (145.3 nm). The topological fluctuations would result of incomplete alteration of crystalline INH to an amorphous structure. In contrast, in-depth visualization of the AgNCs surface showed uniform and homogeneous distribution (Figure S2b).

#### 2.3.5. Particle Size Distribution (PSD), Polydispersity Index (PDI), and Zeta Potential (ZP)

ZP determined the relative stability of AgNPs, and AgNCs (F7, F10, and F13) (Figure S3a–d). The particle size and zeta potential of the AgNCs were found to be (160 ± 2.7 nm; −14.4 mV for F7) (Figure S3b); (208 ± 2.9 nm; −19.8 mV for F10) (Figure S3c); and (281.3 ± 3.6 nm; −19.5 mV) for F13) (Figure S3d). The lowest particle size and the highest ZP were obtained for the formulation F10. This is significantly higher than the values of AgNPs (145.3 ± 2.1 nm, −33.1 mV) (Figure S3a). The PDI of 0.387 within the accepted limited of <1.0 indicates the homogeneity of dispersions. The results revealed the formation of both AgNPs and AgNCs within the nanoscale. In the previous study, a highly stable AgNPs synthesized from garlic extract showed a size lesser than 100 nm with enhanced in vitro antibacterial activity [28]. Furthermore, large particle sizes (>500 nm) can cause toxicity to healthy cells in the body and are not suitable for drug delivery applications [29]. Thus, the PS matters for, at least, some biological activities (Figure S3e–h). Interestingly, Srivatsava and Ahmad reported phytogenic AgNCs prepared from the leaf extract of weed, and the direct conjugation of INH in the extract [30]. The authors showed that the size >1 µm and stability of such nanoconjugates could not be compromised, except in their effectiveness against MDR-TB, as shown through luciferase reported phage (LRP) assay. Further, many studies reported that bactericidal drug encapsulation in AgNPs, due to presence of Silver, may potentiate the bactericidal action of the drug, meantime helping in the reduction of drug dosage [4].

### 2.4. Drug Release Behavior

**Figure 6:**
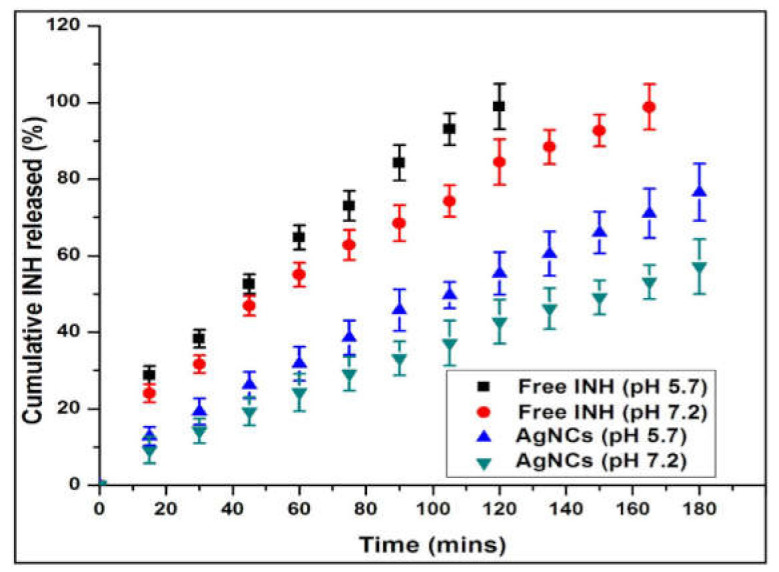
In vitro release of INH from garlic-mediated synthesized AgNCs at 37 °C either at pH 5.7 or pH 7.2. Free INH in 1X PBS was used as control.

The cumulative INH release profiles from AgNCs are shown in Figure 6. The free INH in 1X PBS was used as control. The referred pH of 5.7 and 7.2 was chosen for INH in vitro release because they are like the human physiological skin and lung tissues conditions, respectively [31,32]. The drug release depends on the pH, which is established to be the most significant factor in MDR-TB therapy. The pH of normal tissue varies in function of the pathological process in organs such as lungs and disease-provoked tissues. Free INH release was sustained, and no initial burst release was observed in the two pH conditions, which suggest a potentially beneficial use of the phytogenic AgNPs for topical and intravenous administration. Herein, AgNCs exhibited slower, more sustained and controlled drug release, with the rate of release determined to be half of the times higher than that of free INH in pH 5.7 and lungs environments, respectively. Also, the rate of release of the INH from the AgNCs was half at acidic pH compared to that of pH 7.2. The minor amount of INH released from AgNCs in pH 7.2 is ideal as it then decreases the toxicity of INH to the normal tissue, while the physiological pH of the body can be also maintained at a pH of 7.2 [31]. Similarly, Li et al., reported that the doxorubicin (DOX)-AgNPs displayed slow and controlled release of the drug, with the release rate measured to be three or four times lower than that of free DOX in acidic or neutral environments, respectively [33].

The in vitro INH release from the AgNCs has been well described by Hixon Crowell (0.990 to 0.993) and Table 2 shows the first order (0.976 to 0.994). The model defines the release of drugs in a stable, diffusive state controlled by the particle size and surface area of the formulated AgNCs. Diffusion in a controlled release as free INH at pH 5.7 and 7.4 could control the release rate of INH from AgNCs.

Vieira and co-workers studied that the results obtained for Hixon Crowell are in good accordance with the mechanism of INH release [34]. The kinetic release study showed that release of the drug from the AgNCs in vitro model was the better way to prepare the controlled release of the INH.

### 2.5. Stability

**Figure 7:**
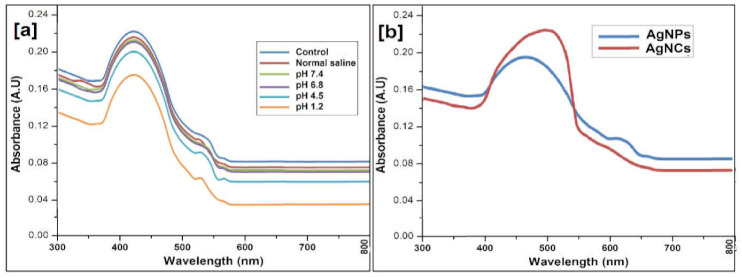
In vitro stability evaluated by UV–Vis spectrophotometry at room temperature for (**a**) AgNCs exposed to 1X PBS for 48 h at indicated pH, and normal saline (0.9% NaCl); (**b**) AgNPs and conjugated with INH.

The stability of AgNPs at various pH of 1X PBS (0.9 % NaCl, pH 1.2, pH 4.5, pH 6.8, or pH 7.4) is shown in Figure 7. Insignificant differences (less than 10%) were detected in the SPR band at 425 nm when the AgNCs were exposed for 48 hrs in the different solutions. After 48 h, about a quarter reduction was observed at pH 1.2 at 425 nm over AgNCs at pH 7.4 (Figure 7a). Thus, the little variation in the position below pH change and the condition of electrolytes revealed a great stability of AgNCs. The reduction in the absorption peak shown in AgNCs due to strong acidic environments (pH 1.2) could increase from an inadequate aggregation of AgNCs as an outcome of the screening of the negative charge on the outer membrane of the AgNPs [35]. AgNPs in normal saline were stable because normal saline mimics the physiological environment (Figure 7b). A structural analysis of INH and AgNPs exhibited their greater binding affinity, as the positive charge of INH have the amino group for contact with AgNPs [36]. This observation anticipated as the INH certainly attached onto the AgNPs at room temperature within 15 min. In addition, this binding efficiency was evaluated by the shift of SPR band i.e., from 425 nm to 442 nm with reference to a higher wavelength (Figure 7b).

### 2.6. Docking Studies

The existing bioactive compounds of garlic were randomly selected to evaluate their extent in antitubercular activity using in silico molecular docking studies. With reference to it, about 23 compounds were selected for the study (Table 3) identified. The stereoisomers of the compounds with low energies were predicted for every ligand and the one which holds the lowest energy 3D structure was reserved.

In addition, the 3D modeled structure (3R6C) of anthranilate phosphoribosyltransferase (trpD) from *Mycobacterium tuberculosis* was obtained from the protein databank (PDB). The protein was prepared using the “protein preparation wizard” tool of Schrodinger suite. After preparation of the protein, a grid was generated through the centroid of active site residues through the van der Waals scaling factor in terms of 1.0 and the partial charge cutoff at 0.25.

The ligand docking was performed with the prepared trpD and the 23 ligands using the “Ligand docking” of Schrodinger maestro. Subsequently, the successful docking, certain properties like as docking score, Glide evdw (Van Der Waals energy), ecoul (Coulomb energy), Glide energy and the interacting residues (Hydrogen bond/ π-π bond) were given precedence and identified. Using these properties, the height of interaction between the protein and the ligands were observed. The predicted respective scores such as docking score, Glide evdw, Glide ecoul, Glide energy and the interacting residues is given in Table 3. The docking score or glide score, which shows the least or lowest score or energy, was considered as the most effective or affinity towards the protein [37].

**Figure 8:**
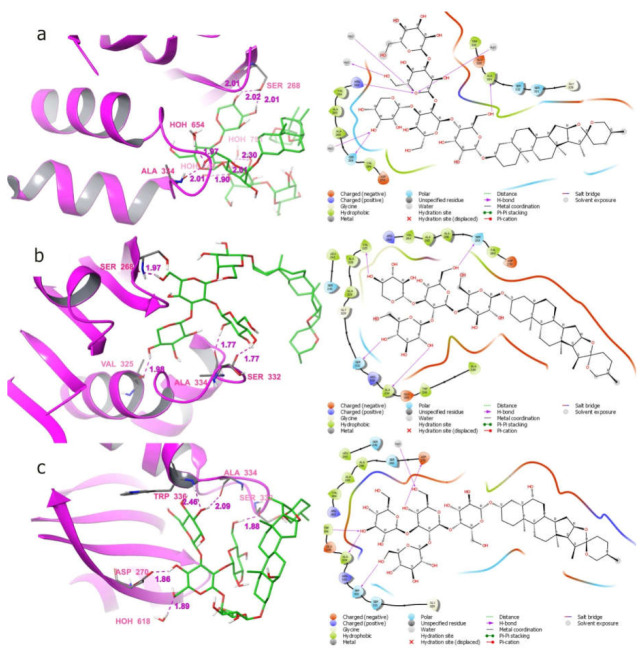

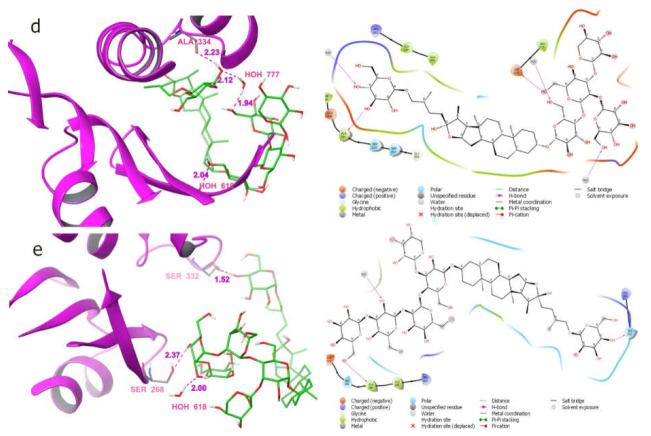
Sativoside R2 (**a**), Degalactotigonin (**b**), and Eruboside B (**c**) interaction map with the protein anthranilate phosphoribosyltransferase (3R6C); Proto-Degalactotigonin (**d**), and Sativoside R1 (**e**) interaction map with the protein Anthranilate phosphoribosyltransferase (3R6C).

The 3D and 2D interaction diagram for the top scored compounds namely Sativoside R2, Degalactotigonin, Proto-desgalactotigonin, Eruboside B and Sativoside R1 were shown in Figure 8.

Using the obtained results (Table 3), most compounds have shown good binding affinity towards the protein trpD. Among them, the compounds Degalactotigonin, Eruboside B, Proto-desgalactotigonin, Sativoside R1 and Sativoside R2, showed the best affinity towards the protein. The compounds Sativoside R2, Degalactotigonin, Proto-desgalactotigonin, Eruboside B and Sativoside R1 have the docking score of −11.04, −10.13, −8.61, −8.05 and −7.1, respectively.

The compound Sativoside R2 had shown well-built hydrogen bond interaction with the amino acid residues ARG263, SER268, ALA334, HOH654 and HOH749 (Figure 8). Also, the Glide evdw, ecoul and Glide energy for Sativoside R2 were −15.81, −17.35, and −33.15, correspondingly. The compound Degalactotigonin with a docking score of −10.13 and observed hydrogen bond interaction with the amino acids such as SER268, VAL325, SER332 and ALA334 (Figure 8). The Glide evdw, ecoul and Glide energy for Degalactotigonin were −30.24, −21.8, and −52.04, subsequently. Furthermore, the compound Eruboside B with the docking score of -8.61 have shown more hydrogen bond interactions with the amino acid residues SER268, ASP270, SER332, ALA334, TRP336, and HOH618 (Figure 8) as well as its Glide evdw, ecoul and Glide energy were −26.07, −18.93, −44.99.

The subsequent top scored compound Proto-desgalactotigonin has the docking score of −8.05, also shown fine hydrogen bond interaction with the residues ASP270, LEU272, ALA334, HOH618, and HOH777 (Figure 8). The Glide evdw, ecoul and Glide energy of Proto-desgalactotigonin were −24.65, −17.87 and −42.52. The following compound Sativoside R1 also shown hydrogen bond interaction with the amino acid residues ALA266, SER332, HOH618 and the Glide evdw, ecoul and Glide energy were −7.55, −13.43 and −20.99, correspondingly (Figure 8).

Furthermore, the other compounds also showed excellent hydrogen bond interactions with less binding affinity with the protein. Consequently, the compounds by means of its donor or its acceptor side chains/groups form hydrogen bonds with residues are expected to encompass superior binding affinity. Amongst the 23 compounds two compounds namely Protoeruboside B and Sativoside B1 have not shown any binding properties with the protein as shown in Figure 8.

Taken together, the highlighted top scored compounds based on the docking score against the tubercular marker were in the order of Sativoside R2 > Degalactotigonin > Proto-desgalactotigonin > Eruboside B > Sativoside R1. These compounds could be tested, ex vivo and in vivo, alone or in combination of INH, and loaded (or not) into AgNPs or AgNCs (INH-loaded AgNPs) for anti-MDR-TB activity.

## 3. Materials and Methods

### 3.1. Reagents

Isoniazid hydrazide (INH) was purchased from Sisco Research laboratories Pvt. Ltd., (Maharashtra, India). Silver Nitrate (AgNO_3_) was obtained from Alpha Chemika (Mumbai, India). The bulbs of Allium sativum (garlic) were provided from the local market of Tricirappalli (Tami Nadu, India).

### 3.2. Preparation of Garlic Extracts

The garlic cloves were peeled and washed with water filtered, using a 0.22 µm membrane filter (Sigma-Aldrich Chemical Pvt. Ltd., Bangalore, India), to remove any dust/particulate matter present on them. The aqueous extract was then prepared by grinding 10 g of garlic cloves with 100 mL of Millipore milliQ water using mortar and pestle. The extract was then filtered using Whatman filter paper (11 µm), and the filtrate was stored at 4°C for further use [38].

### 3.3. Green Synthesis of Silver Nanoparticles (AgNPs)

For the reduction of silver ions, 1 mL of freshly prepared aqueous garlic extract was added to 19 mL of 0.1 M AgNO_3_. This reaction mixture was continuously stirred (650 rpm) using magnetic stirrer (REMI-2MLH, Maharashtra, India) and exposed to the bright sunlight (summer, 40.9 °C, in Tiruchirappalli, Tamil Nadu, India, in the month of April 2021; dry air condition). Within few seconds (less than 5 s) of exposure to the light, the colorless solution started changing to yellow-brown indicating the formation of a silver colloid [39]. The intensity of color increased with increasing time, and reached plateau after 15 min. This was observed under UV-Vis spectrum at 433 nm. The synthesized AgNPs were purified by washing them thrice with distilled water followed by centrifugation at 2500 rpm for 20 min before their resuspension in distilled water.

### 3.4. Preparation of Isoniazid Hydrazide (INH) Silver Nanoconjugates (AgNCs)

Different quantities of saturated solutions of INH and AgNPs were considered, as shown in Table 4. Both were mixed simply, allowed to sonicate (Sonics & Materials VCX 750, Newtown, CT, USA) for 10 min (40% amplitude and pulse rate 5/3 s) and kept in microbial incubator for 1 h at 37 °C [40]. Subsequently, each sample was taken out and centrifuged at 4000 rpm for 30 min (Eppendorf centrifuge 5430R). Each pellet was collected and dried in a vacuum oven for 2 h at −80 Hg mm (T-M Vacuum, Cinnaminson, NJ, USA). The prepared isoniazid silver nanoconjugates (AgNCs) were analyzed using UV-Vis spectrometer (Agilent Cary, USA) at the range wavelength of 200–800 nm.

### 3.5. In Vitro Characterizations

The raw materials (i.e., INH and AgNPs) and the freshly prepared AgNCs were characterized for drug content, FTIR, UV-Vis spectroscopy, XRD, HR-TEM, SEM, EDX, PS, and ZP, according to methods previously published by our previous works [41,42,43].

#### 3.5.1. Drug Entrapment Efficacy (EE) and Loaded Capacity (LC)

The amount of INH encapsulated into AgNCs was estimated by quantifying the amount of INH released from AgNCs upon sonication. Briefly, AgNCs was centrifuged at 15,000× *g* and 4°C for 30 min. Then, the top layer of unentrapped INH was analyzed using UV-Vis spectrophotometry (*n* = 3). A calibration curve of INH at the concentration range of 1 to 6 µg/mL was plotted using MilliQ water for the estimation of the drug content [44]. The drug EE (%) and LC (%) were calculated by the following Equation (1) and (2).

EE (%) = (Experimental INH content)/(Theoretical INH content) × 100(1)

DL (%) = (Weight of INH)/(Weight of INH-AgNCs) × 100(2)

#### 3.5.2. In Vitro Drug Release Behavior

The in vitro drug release study was done at 37 ± 0.5 °C under continuous stirring at 100 rpm in 100 mL of either sodium phosphate buffer solution (PBS, 1X, pH 5.7), or simulated lung fluid from buffer medium at pH 7.2. At zero-time interval, samples were placed into the beaker and 5 mL of samples were pumped out and filtered using Whatman filter paper (11 µm) every 15 min for 5 hrs. The INH dissolved in the medium was determined by UV-Vis spectrophotometry (425 nm). A correction was applied for the replacement of the medium to maintain the sink condition [45]. The cumulative drug release was calculated using by the following Equation (3).

Release (%) = (Released INH)/(Total INH in AgNCs) × 100(3)

To assess the kinetic modeling, the dissolution data of the best formulation (F13) in pH conditions (pH 5.7 and pH 7.2) were incorporated to various mathematical models (Table 5), like zero order, first order, Higuchi, Korsmeyer-Peppas models to establish the kinetics of drug release [46].

#### 3.5.3. In Vitro Stability Studies

Briefly, 1 mL of AgNCs were treated with 0.5 mL of each normal saline (0.9% NaCl *w/v*) and PBS 1X adjusted at pH ranging from pH 1.2 to pH 7.4. The solution was incubated at 37 ℃ for 48 h and were analyzed spectrophotometrically [47].

### 3.6. Phytochemical Analysis

**Figure 9:**
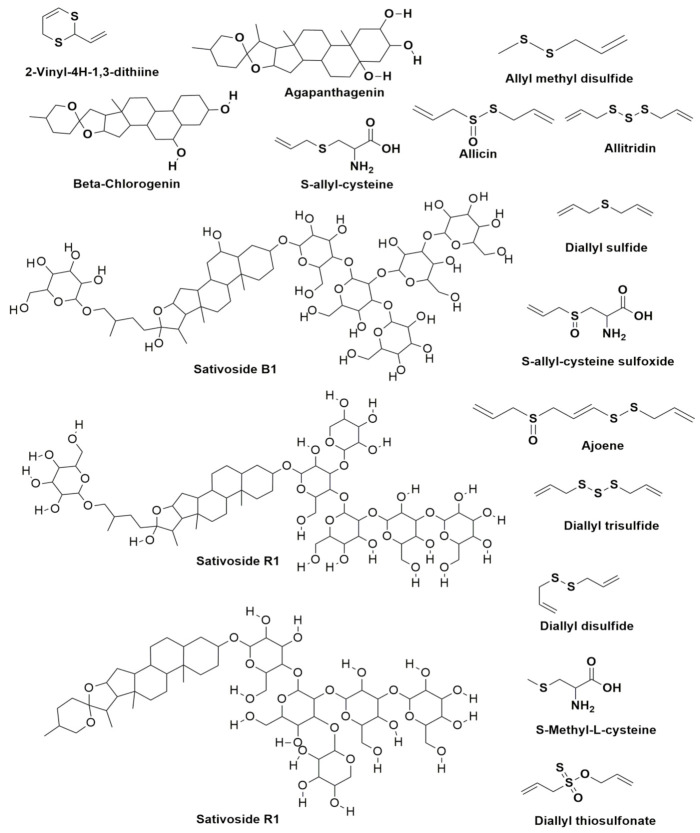
The bioactive compounds from Allium sativum (garlic) and their chemical structures.

**Figure 10:**
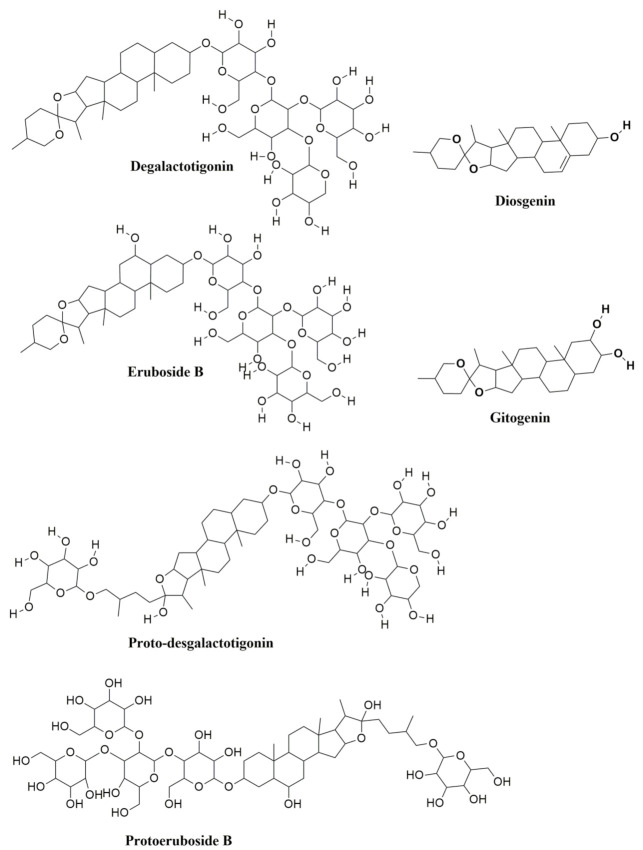
The bioactive compounds from Allium sativum (garlic) and their chemical structures.

The phytochemical or bioactive constituents isolated from garlic were chosen randomly to support our study. A list of recognized 23 phytochemical compounds through the literature was given in Table 5 [48,49,50] and were examined for their anti-TB activity using molecular docking study. The structures of the elected compounds were shown in Figure 9 and Figure 10. The docking study was performed with the 23 compounds against the protein Anthranilate phosphoribosyltransferase (trpD) from *Mycobacterium tuberculosis* (complex with inhibitor ACS179-PDB: ID 3R6C) using the Schrodinger suite 2020-1 software.

### 3.7. Docking Study

#### 3.7.1. Ligand Preparation

The script designed in LigPrep module offer well-organized tools for the preparation of ligands collectively and constantly [51]. It also prepares first-rate three-dimensional (3D) molecular structures and creates single low energy 3D structures with accurate chiralities. It verifies the ionization states, tautomers, stereochemistries, ring conformations of the ligands. It eradicates the molecules using a wide range of criteria such as molecular weight or specific numbers as well as the presence of functional groups. The acknowledged 23 compounds of garlic were drawn using the Chem office tool and saved in .sdf format and imported in to LigPrep module of Schrodinger. The two dimensions (2D) structures of the compounds were corrected into three dimensions (2D) for the docking studies (Figure 9 and Table 3). The force field OPLS-2005 [52,53] was utilized for the geometrical optimization by condensed newton conjugate gradient (TNCG) minimization. The ligands are prepared using the LigPrep (Schrödinger Release 2020-1, 2020) with Epik [54] by 7 ± 2.0 pH units to add up protonation as well as tautomeric states using the force field OPLS2005. The acknowledged 23 compounds of garlic were drawn using the Chemoffice tool and saved in .sdf format to be imported into the LigPrep module of Schrodinger [55,56].

#### 3.7.2. Protein Preparation and Receptor Grid Generation

The 3D crystal structures of anthranilate phosphoribosyltransferase (trpD) from *Mycobacterium tuberculosis* (complex with inhibitor ACS179) were retrieved from the Protein Data Bank database (PDB: ID 3R6C) [57]. The Glide utilizes a script named “all-atom force field” for precise energy evaluation. Glide also align, allocate proper bond orders and ionization states. It reorganizes the missing side chains with reassured steric clashes. The intact process was performed using the Protein Preparation Wizard (PPW) module of Schrodinger suite.

Each set of the receptor fields were symbolized as a grid by a shape and property which steadily afford extra precise scoring of the ligand pose [58]. The choice in each tab of the receptor grid generation panel helps to describe the structure of the receptor by excluding few co-crystallized ligands if present. It also assists to identify the spot and figure of the active site as “receptor grids” to set up Glide constraints [59].

#### 3.7.3. Ligand Docking

The ligand docking process stands in the need of previously designed receptor glides and a single or multiple ligand structure [60]. Glide uses an E-model scoring function to identify the best protein ligand complexes for a ligand and the Glide score function to rank the compounds to differentiate the strong bonding of ligand (active) and the less bonding (inactive) with the proteins [61]. The least scoring compound is considered as the most effective compound against the protein. Herein, the Glide XP (extra precision) module of Schrödinger Suite was used for the ligand docking study. The glide or docking score of the ligand helps to discriminate the molecules as per their interacting capability.

### 3.8. Statistical Analysis

The data were expressed by mean ± standard deviation (STD) from three independent experiments. ANOVA (OriginPro 2021b trial version; USA) was applied to compare the data. *p* < 0.05 was kept as significant.

## 4. Conclusions

To the best of our knowledge, the present work reports for the first time the green synthesis of size adjusted AgNPs using a garlic extract in which the anti-TB INH was encapsulated. The applicability of these AgNPs as carriers for the transport of cationic drugs, was demonstrated by successfully loading INH into the synthesized AgNPs. The prepared phytogenic AgNPs were spherical, relatively small, crystalline in nature, and showed excellent stability under various physiological conditions. The adequate encapsulation of INH into AgNPs was confirmed through UV-Vis spectrum and FTIR analyses. The sustained and controlled release of the INH release rate was calculated to be one-half the release rate of free INH in acidic and neutral environments. The combination of garlic extract derived synthesis of AgNPs and the conjugation with INH would pave the way for the successful treatment against MDR–TB. However, future studies are required to confirm their effectiveness against drug resistance strain of *Mycobacterium tuberculosis* through LRP assay and/or other suitable ex-vivo and in vivo methods (cytotoxicity and hemocompatibility studies, disk-diffusion assay, animal model) for its appropriate drug delivery applications. Importantly, by using the technique of molecular docking, the elected structures of garlic were analyzed for their binding modes against the protein anthranilate phosphoribosyltransferase (trpD) from *Mycobacterium tuberculosis*, which shown important function against tuberculosis. The compounds Sativoside R2, Degalactotigonin, Proto-desgalactotigonin, Eruboside B, and Sativoside R1 were the best-docked compounds for the marker trpD. Further extensive studies are required to confirm their computational results. These compounds may be considered as a choice in designing novel gifted molecules and (nano)-formulations which would help to inhibit trpD.

## Figures and Tables

**Table 1:** The FTIR spectrum wavenumbers and its concern bands of samples.

Sample	Wavenumber (cm^−1^)	Band (cm^−1^)	Characteristic Function
Garlic Extracts	3455.81, 2884.02, 1644.02, 1407.78, 1132.97, 1032.69, 932.414, 617.109, 490.795	3584–3700	–OH Stretching
AGNPS	3778.83, 2962.13, 2885.95, 2808.81, 1579.41, 1394.28, 1087.66, 840.812, 615.818, 485.974	2800–3000	N-H Stretching
INH	3774.01, 3665.05, 3304.43, 3235, 3113.51, 3002.62, 2873.42, 2664.18, 1957.39, 1669.2, 1554.34, 1409.71, 1330.64, 1217.83, 1135.87, 1057.76, 995.089, 889.023, 844.669, 745.352, 668.214, 499.473, 434.869	2850–2975	Alkane Group
F7	3677.59, 3428.81, 3331.43, 3055.66, 2998.77, 2946.7, 2963.56, 2884.99, 2836.77, 2818.45, 2766.39, 2676.71, 2290.05, 1780.94, 1701.87, 1557.24, 1497.45, 1426.1, 1327.75, 1275.68, 1211.08, 1025.94, 883.238, 771.387, 706.783, 674.963	1210–1163	C=O Stretching
F10	3677.59, 3427.85, 3331.43, 3219.58, 3053.73, 2939.95, 2887.88, 2830.99, 2673.82, 1854.22, 1753.94, 1702.84, 1555.31, 1496.49, 1424.17, 1326.79, 1209.15, 1036.55, 883.238, 772.351, 672.071	700–900	C-H Bending
F13	3697.84, 3430.74, 3013.23, 2931.27, 2886.92, 2819.42, 2675.75, 2529.18, 1878.33, 1752.01, 1698.98, 1550.49, 1495.53, 1424.17, 1327.75, 1210.11, 1040.41, 880.345, 783.922, 673.035, 456.082	730–665	C=C Bending

**Table 2:** In vitro release kinetics of SLN (Data are expressed as mean ± SD, *n* = 3).

Correlation Coefficient (r^2^)						
F #13	Zero-Order	First Order	Higuchi	Hixon Crowell	Korsmeyer-Peppas	Release Exponent (*n*)
Free INH (pH 5.7)	0.965 ± 0.13	0.976 ± 0.36	0.984 ± 0.31	0.991 ± 0.12	0.88 ± 0.19	7.8 ± 0.32
Free INH (pH 7.2)	0.957 ± 0.13	0.994 ± 0.36	0.986 ± 0.31	0.990 ± 0.15	0.89 ± 0.18	6.4 ± 0.32
AgNCs (pH 5.7)	0.986 ± 0.17	0.994 ± 0.22	0.964 ± 0.33	0.993 ± 0.16	0.834 ± 0.27	4.98 ± 0.67
AgNCs (pH 7.2)	0.841 ± 0.17	0.993 ± 0.22	0.965 ± 0.33	0.991 ± 0.18	0.950 ± 0.22	0.115 ± 0.67

F: Formulation; INH: Isoniazid hydrazide; AgNCs: Silver nanoconjugates (AgNPs + INH).

**Table 3:** The docking score, Glide evdw (Van Der Waals energy), ecoul (Coulomb energy), interacting residues and the type of interaction of 23 bioactive compounds with the protein anthranilate phosphoribosyltransferase (trpD). The docking scores calculated using Glide program of Schrodinger Maestro (version 2018.1).

Target Protein	Name of the Compound	Docking Score	Glide Evdw	Glide Ecoul	Glide Energy	Interacting Residues (HB/Pi-Pi)
3R6C	2-Vinyl-4H-1,3-dithiine	−2.99	−22.86	−1.82	−24.67	-
	Agapanthagenin	−3.99	−20.09	−6.95	−27.03	ALA334
	Ajoene	−1.86	−22.85	−5.86	−28.71	HOH749
	Allicin	−2.37	−17.87	−8.36	−26.23	HOH749
	Allitridin	−0.99	−17.81	−1.40	−19.21	-
	Allyl methyl disulfide	−1.98	−18.93	−1.39	−20.31	-
	Beta-Chlorogenin	−3.30	−28.67	−2.28	−30.96	HOH618
	Degalactotigonin	−10.13	−30.24	−21.80	−52.04	SER268, VAL325, SER332 and ALA334
	Diallyl disulfide	−1.64	−21.25	−2.02	−23.27	-
	Diallyl sulfide	−1.10	−18.85	−1.72	−20.57	-
	Diallyl thiosulfonate	−2.40	−23.11	−4.31	−27.42	ARG263
	Diallyl trisulfide	−0.99	−17.81	−1.40	−19.21	-
	Diosgenin	−2.60	−21.36	−4.09	−25.45	ALA334 and HOH618
	Eruboside B	−8.61	−26.07	−18.93	−44.99	SER268, ASP270, SER332, ALA334, TRP336 and HOH618
	Gitogenin	−3.27	−22.20	−3.98	−26.17	ALA334 and HOH618
	Proto-desgalactotigonin	−8.05	−24.65	−17.87	−42.52	ASP270, LEU272, ALA334, HOH777 and HOH618
	Protoeruboside B	-	-	-	-	-
	S-allyl-cysteine sulfoxide	−3.15	−16.07	−12.32	−28.39	ARG263, ALS334, TRP336, HOH749, and HOH777
	S-allyl-cysteine	−2.80	−15.19	−13.07	−28.26	ALS334, GLU335, TRP336, HOH749 and HOH777
	Sativoside B1	-	-	-	-	-
	Sativoside R1	−7.10	−7.55	−13.43	−20.99	ALA266, SER332 and HOH618
	Sativoside R2	−11.04	−15.81	−17.35	−33.15	ARG263, SER268, ALA334, HOH654 and HOH749
	S-Methyl-L-cysteine	−2.48	−13.25	−11.01	−24.26	ARG263, ALA334, TRP336 and HOH749

HB—Hydrogen bonding; Pi-Pi—π-π bond.

**Table 4:** λmax and absorbance values of AgNCs at various compositions (*v/v*).

#F	AgNCs		Trial 1		Trial 2		Trial 3	
-	INH (μL)	AgNPs (μL)	λmax	Abs	λmax	Abs	λmax	Abs
1	100	0	262	0.020	263	0.031	261	0.031
2	0	100	415	0.016	413	0.018	412	0.015
3	100	100	266	0.024	261	0.029	259	0.098
4	100	900	274	0.051	343	0.019	259	0.060
5	200	800	355	0.015	356	0.020	259	0.057
6	300	700	259	0.051	259	0.051	383	0.042
7	**400**	**600**	**271**	**0.034**	**269**	**0.031**	**271**	0.045
8	500	500	270	0.055	261	0.034	352	0.026
9	600	400	260	0.043	259	0.038	261	0.132
10	**700**	**300**	**261**	**0.025**	**263**	**0.057**	**263**	0.036
11	800	200	264	0.025	267	0.044	259	0.041
12	900	100	264	0.049	341	0.024	259	0.078
**13**	**1000**	**1000**	**264**	**0.053**	**265**	**0.028**	**264**	**0.031**

F: Formulation; INH: Isoniazid hydrazide; AgNPs: Silver nanoparticles; AgNCs—Silver nanoconjugates; λmax: Maximal wavelength; Abs: Absorbance.

**Table 5:** List of bioactive compounds present in Allium sativa (garlic).

	Name of the Compound	PubChem/ACS/Fooddb		Name of the Compound	PubChem/ACS/Fooddb
1	2-Vinyl-4H-1,3-dithiine	133337	13	Diosgenin	99474
2	Agapanthagenin	15558507	14	Eruboside B	13787750
3	Ajoene	5386591	15	Gitogenin	441887
4	Allicin	65036	16	Proto-degalactotigonin	14464370
5	Allitridin	16315	17	Protoeruboside B	FDB003677
6	Allyl methyl disulfide	62434	18	S-allyl-cysteine sulfoxide	15558642
7	Beta-Chlorogenin	10717615	19	S-allyl-cysteine	9793905
8	Degalactotigonin	162401	20	Sativoside B1	14464368
9	Diallyl disulfide	16590	21	Sativoside R1	131752731
10	Diallyl sulfide	11617	22	Sativoside R2	3474285
11	Diallyl thiosulfonate	88093432	23	S-Methyl-L-cysteine	24417
12	Diallyl trisulfide	16315			

## Supplementary Materials

Figure S1: EDX spectrum of AgNPs synthesized from *Allium sativum* (garlic); Figure S2: SEM images of INH-AgNCs at (a) bar scale of 2 μm, (b) bar scale of 5 μm (magnification ×2.5); Figure S3: Zeta potential of (a) AgNPs, (b) F7, (c) F10, and (d) F13. Zeta particle size distribution of (e) AgNPs, (f) F7, (g) F10, and (h) F13; Figure S4: Zeta particle size distribution of (a) AgNPs, (b) F7, (c) F10, and (d) F13.

## Sample Availability

Samples of the compounds are not available from the authors.
