# Supplementary material for: Superfast Synthesis of Stabilized Silver Nanoparticles Using Aqueous Allium sativum (Garlic) Extract and Isoniazid Hydrazide Conjugates: Molecular Docking and In-Vitro Characterizations
Source: Molecules. 2021 Dec 24;27(1):110. doi: 10.3390/molecules27010110 (PMC8746848; doi:10.3390/molecules27010110)
Supplement: Supplementary file 1 [file molecules-27-00110-s001.zip › molecules-1510824-supplementary.pdf]

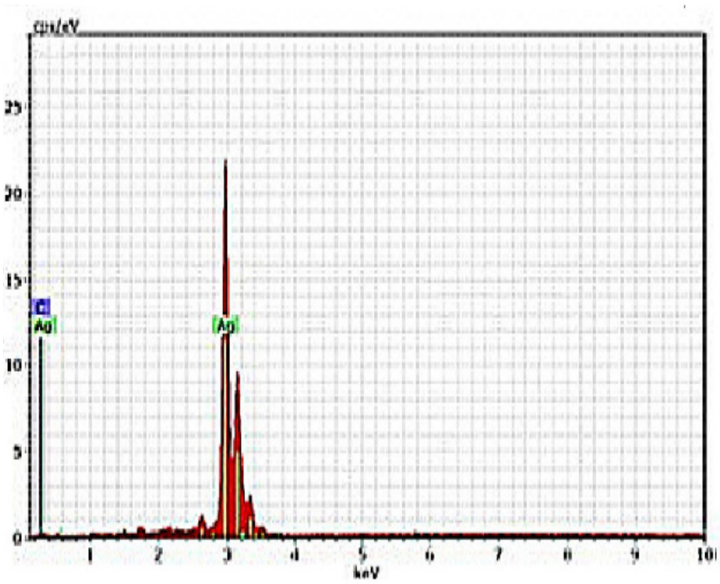

Figure S1. EDX spectrum of AgNPs synthesized from *Allium sativum* (garlic).

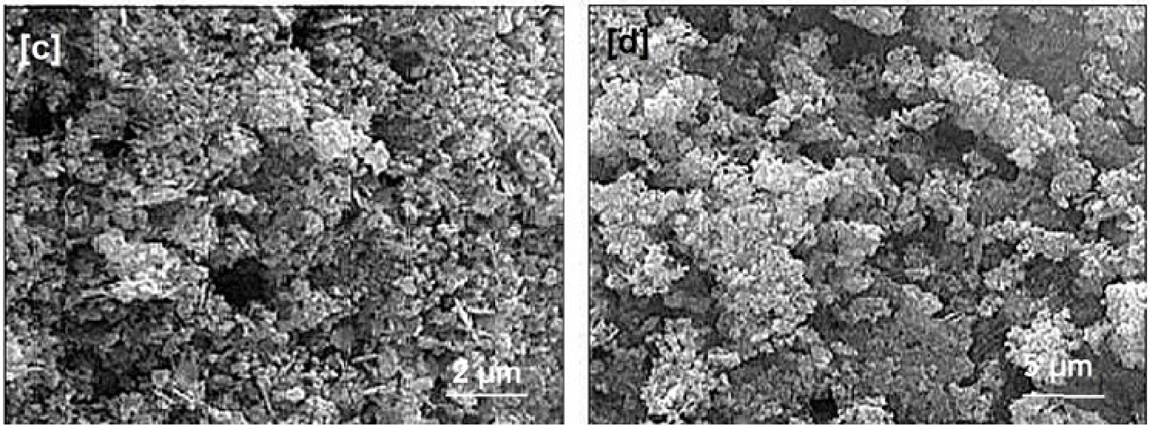

Figure S2. SEM images of INH-AgNCs at **(a)** bar scale of 2 nm, **(b)** bar scale of 5 m (magnification x2.5).

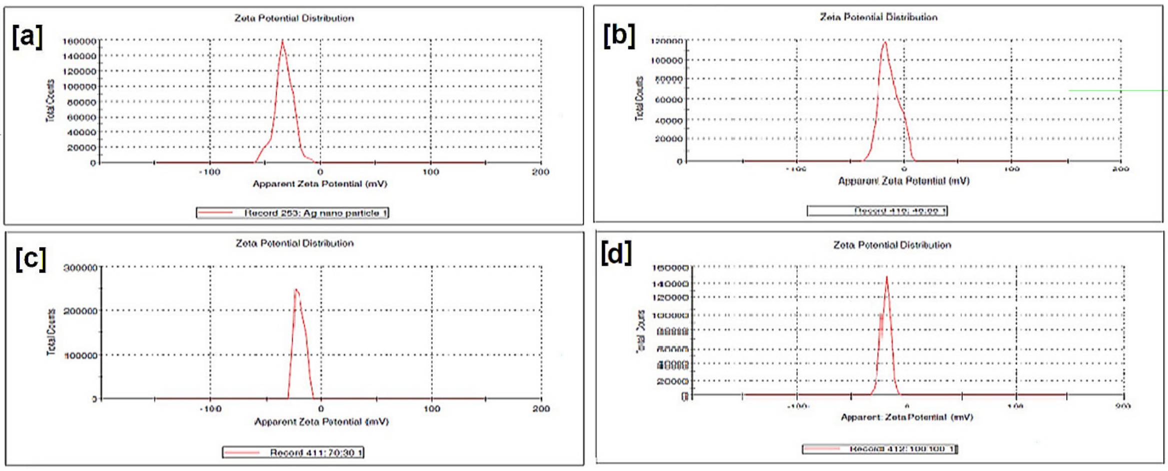

Figure S3. Zeta potential of **a)** AgNPs, **b)** F7, **c)** F10, and **d)** F13.

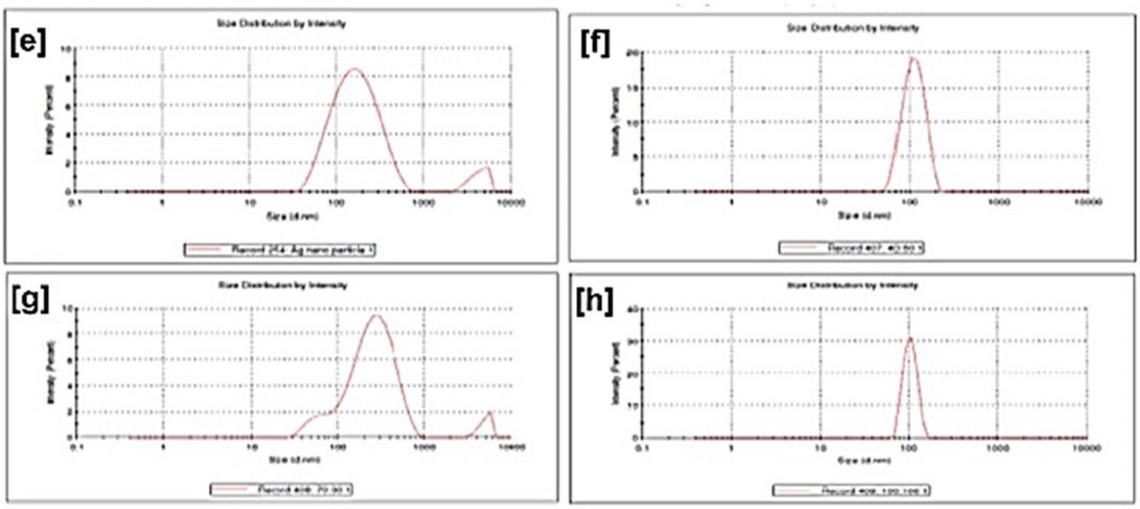

Figure S4. Zeta particle size distribution of **a)** AgNPs, **b)** F7, **c)** F10, and **d)** F13.
